# From smoking guns to footprints: mining for critical events of toxicity pathways in transcriptome data

**DOI:** 10.1007/s00204-015-1497-6

**Published:** 2015-04-08

**Authors:** Jörg Rahnenführer, Marcel Leist

**Affiliations:** 1 Department of Statistics, TU Dortmund University, Dortmund, Germany; 2 Doerenakmp-Zbinden Chair for In Vitro Toxicology and Biomedicine, University of Konstanz, PO Box M657, 78457 Konstanz, Germany

Toxicological studies can be designed in very different ways, and the 2007 suggestion of the National Academy of Sciences of the USA has shown that the mode of working of a whole field of science can be reconsidered (Blaauboer et al. 2012; Leist et al. 2008, 2012, 2014). As study design often heavily influences or even drives the type of outcome, it may pay off well to invest some thought about how science is structured in most reports. Two extremes of a wide spectrum are considered here for simplification, the frog’s and the eagle’s perspective. The former one is the classical hypothesis-driven study that looks at the object of interest from very close by. In fact, the observer looks almost from below at her/his target, e.g. a favourite protein, and studies a specific feature, e.g. ubiquitination, with very high local resolution. During this type of research, most effort is invested in confirming a certain hypothesis that had been presented to granting bodies, in some cases years before the study even started. A danger with this approach, which predominates biological research at the moment, is that the context may get lost and that it often remains unclear, how broadly the highly detailed results may apply. Moreover, it is generally difficult to piece the individual localized findings together to more general conclusions. Finally, the types of hypotheses that are being tested are typically biased towards prior knowledge.

The opposite extreme of studies provides descriptive data only and has become particularly popular in modern data-rich scenarios. Rather than being hypothesis based, this approach of an unbiased, as complete as possible description of a cell response, can help to generate entirely new hypotheses. For obtaining a broad overview, a rather large observational distance is chosen. Thus, detail and resolution may be lost, if broad context is to be covered, and some observations may become so blurry that they are of no use.

There are many ways to combine such extremes of study approaches to the benefit of science. For instance, unbiased screens may be followed up by detailed mechanistic confirmations of a hypothesis or of findings derived from the screen. Or, a general description of transcriptome responses may be followed by the identification of gene regulatory networks that drive such responses. This latter type of combined approach is still relatively rare in toxicology, and a particularly interesting example has been presented by Maertens et al. (2015), who undertook the second step, on the basis of data already published and available in the public domain after the first step had been taken (Miller et al. 2004). Their approach demonstrates particularly well that additional mileage can be gained, on the basis of the same data, if new exploratory statistical and bioinformatics methods, algorithms and tools are used.

The Maertens study takes its starting point from the question how 1-methyl-4-phenyl-tetrahydropyridine (MPTP) exerts its toxicity. This experimental neurotoxicant is extremely well studied (more than 3000 publications), and some features of its toxicity pathway are well established. For instance, the compound is metabolized in brain to 1-methyl-4-phenylpyridinium (MPP+), and this metabolite is transported through the dopamine transporter into dopaminergic neurons of the *S. nigra*, where it blocks complex I of the mitochondria. This leads to the generation of reactive oxygen species (ROS) and the impairment of ATP production, and eventually to cell death. However, neurons can survive well the ATP reduction due to the direct inhibitory effects of MPP+ (Pöltl et al. 2012; Schildknecht et al. 2009; Krug et al. 2014), and it is not clear why cells in brain die after several days from ROS triggered by MPP+, even though the compound is washed out from the brain within hours (Cui et al. 2009). It is also unclear how the protection by cyclooxygenase inhibitors fits this sequence of events, why only some of the neurons die and what role the pronounced transcript alterations, peroxynitrite formation (Schildknecht et al. 2011), or mitochondrial fissions (Rappold et al. 2014), play in cell death. A quantitative or, at least, semi-quantitative adverse outcome pathway has not yet been established for MPTP toxicity, although this is possibly the best-characterized experimental compound triggering neurotoxicity from animal to man.

A screening approach to gain more information would be to obtain transcriptome data from MPTP-treated mice or from human neurons treated with MPP+. Such data can be explored in different ways. The first level would use standard statistical methods to identify differentially expressed genes and possibly some gene signatures that distinguish different time points or doses or that separate MPTP responses from other toxicant responses. Usually, also overrepresented gene ontology terms are identified amongst the groups of co-regulated genes, or genes are clustered according to their statistical co-regulation behaviour (Krug et al. 2013b; Grinberg et al. 2014; Zimmer et al. 2012; Balmer et al. 2012). This is where most studies stop, and the Martens study went further. In general, the next level switches attention from individual genes to gene networks and superordinate biological processes. The architecture of the transcriptome response is studied (Waldmann et al. 2014) with the intention to identify underlying pathways and transcription factors (Krug et al. 2013b), or rather transcription factor networks controlling the processes or giving evidence of cellular signalling events.

However, there is an apparently serious conceptual problem linked to this: key factors may not change their transcript level, as they may be regulated by phosphorylation or by post-transcriptional control of their protein level. Moreover, critical events may not occur on transcript level, but rather affect metabolism (Latta et al. 2000), protein phosphorylation (Selenica et al. 2007), cell morphology/function (Krug et al. 2013a; Zimmer et al. 2014; van Vliet et al. 2014) or classical cell signalling events (cAMP, Ca2 + alterations). Does this limit the usefulness of transcriptome analysis for pathway identification? To answer this, one may look at the study strategy in various other disciplines: geology, astronomy, history and paleontology. Scientists in these fields can hardly ever directly observe events of interest—there are no smoking guns that directly indicate who shot the sheriff. Still, these respectable disciplines have constructed impressive mechanistic/causal chains of events, not so different from a toxicity pathway that explains how a molecular initiating event triggered by a toxicant links to its adverse outcome for an affected person. The solution in these other fields is based on following footprints instead of following the lion directly. Sometimes even footprints of footprints are sufficient: an eagle’s view observer would get a pretty good idea on where the lions are by observing the behaviour of prey and scavengers such as jackals and vultures. This approach can also be translated to transcriptome data. Transcriptome analysis cannot measure oxidative stress, but it can very clearly detect its footprints, in the form of Nrf-2 target genes (Fredriksson et al. 2014; Limonciel et al. 2015; Wilmes et al. 2014; Hamon et al. 2014). For instance, a recent study identified ATF-4 as master regulator of cell adaptations to MPP+, although ATF-4 was not identified in the transcriptome data set directly (Krug et al. 2014). The same applies to the study of Maertens. The transcription factor SP-1 was identified as a main hub in a regulatory network, orchestrating the MPTP response, although SP-1 itself was not amongst the regulated genes.

This is an important lesson learnt from this study. But how was this achieved, and what steps need to be taken for such an approach? Two problems need to be addressed: the first is how to deal with noise in the data. A standard approach is to work only with information far beyond the signal-to-noise limit, typically with differentially expressed genes that are both highly significant and strongly regulated. This procedure misses all real signals that are weak. This is a problem, since sometimes several weak, but coordinated signals can have large consequences. The second issue is how to select biologically meaningful information from everything that could be statistically meaningful. Already, a dozen regulated genes can form billions of patterns and gene regulatory networks. Therefore, just finding the factors, i.e. the nodes of a network, is often of little use. One promising solution to both issues is to combine the worlds of the eagle and the frog, by sequentially using different types of approaches; for instance an initial unbiased statistical method with high sensitivity and low specificity is overlapped with biological data to filter information and to provide specificity. More such layers may be added. Even biased steps relying on expert knowledge may provide starting points that may then be further confirmed by non-biased methods (Zimmer et al. 2011; Kuegler et al. 2010). The power of using additional biological information to enhance results obtained from purely statistical analyses has not been leveraged sufficiently to provide toxicological information, as this requires interdisciplinary work and combining very different types of expertise.

The study of Maertens used a series of statistical and biological methods and tools in the following way: starting point was a set of transcriptome data from mouse brain tissue, with four replicates each from day 0, day 1 and day 7 relative to MPTP dosing. The data were used for weighted gene correlation network analysis (WGCNA), i.e. a systems biology tool that allowed clustering of correlated genes. Thus, this first step was based on finding groups of genes (so-called modules) that were similar to one another concerning the time course of their regulation. The background consideration was that such co-regulated genes have a higher likelihood to be involved in common biological functions.

This initial statistical step yielded 1247 genes arranged in five clusters. Such clusters were formed by groups of genes with stronger connectivity (with a mathematically defined threshold) to one another than to the genes outside the cluster. For the next steps of the work, the grouping of the genes within these clusters (=modules) was important. (The relationships between genes in a module were considered only in later steps.) For instance, gene ontology term overrepresentation was studied for the genes of the clusters, as conventional type of approach.

As next step, the study went beyond this very common form of analysis, and the clusters were analysed against the MSigDB database. This approach allowed for the search of many overrepresented features. Here, work concentrated on the overrepresentation of transcription factor (TF) binding sites (TFBS) in the promoters of the genes in the cluster, and for each of the five clusters, binding sites for more than 10 TFs were found to be overrepresented. Such information can be interesting, if several related situations are compared, but it yields very few answers to the initial biological question. Therefore, the next step provided a focus by incorporation of biology background data. It involved text mining of the biological literature, as all identified TFs were investigated (screening of MEDLINE entries). They were retained for further analysis, if more than two publications were found connecting the TF to either Parkinson’s disease or MPTP. On this basis, two interesting steps were taken to arrive at a new level of information: (1) the TF identified for each cluster during the previous steps was added to the group of genes belonging to that cluster. (2) Then, this slightly larger set of genes was used again for network construction.

However, the way this network was built differed from the WGCNA, in that existing biological information was used for its construction, instead of statistical correlations. The information was derived from the FANTOM4 database that contains multiple layers of information on gene interactions. Some are derived from experimental studies, such as siRNA knockdown or chromatin immunoprecipitation (ChIP, a method to physically map TF binding sites). The latter two types of information were chosen to build a gene regulatory network (GRN), in which not only information on connections is given, but also information on the directionality of the connection (i.e. A regulates B, instead of A is connected to B). Within this new network, the component with most connected genes was identified and considered as biologically important regulation module. A variation of this approach was building the GRN without the TF added. This ‘control experiment’ showed that there were dramatically less connections found in the largest component. Finally, the process of building a GRN was repeated for the entire pool of genes from all five clusters (plus the identified TF), in order to identify important hubs (nodes with particularly many connections), not just for one cluster, but also as links between clusters. This network consisted in the end of 256 genes, and SP1 emerged as the major hub in the network of most clusters (while it was not evident from the pure correlation analysis, that SP1 could play any role).

Thus, the study suggests approaches (only one of many is demonstrated) that combine various existing data to generate new hypotheses on toxicity networks. It might be an incentive for enhanced data mining efforts, with toxicologists, biostatisticians and systems biologists closely collaborating to form a discipline of systems toxicology. There is still hesitation, whether really new information can be gained from this. An analogy may help to start the thought process. Returning to the ‘eagle’s view’, we might observe a city’s traffic from very high above, continuously, or in the form of snapshots, like a transcriptome study. Many reasons for large disturbances will be easy to observe directly: a traffic accident, a construction site, a street demonstration, etc. For others, we will only see the footprints, left in the traffic: signals by traffic lights, we cannot see from above. Traffic lights do not change in quantity and location, they only change their functional state, and this cannot be observed from above. Still, a good enough analysis of the traffic flow will give very exact information on where they are and which role they play. We can predict their behaviour individually, or in coordination as ‘phased traffic lights’, and we can understand them as a cause for systems breakdown, when they stop working. This may be a farfetched comparison, but it appears to be worth giving it a try to explore how far it holds.

Such confirmation approaches will need to go one step further in the end. Ultimately, the circle of arguments needs to be closed by a biological confirmatory step in a similar model system, and attempts are needed to confirm such findings in related other model systems. Questions also need to be asked about the internal consistency of the data. For instance, the starting point of the study was a time-wise correlation of transcript responses. The follow-up question would be whether new hubs and networks follow a plausible time course. For instance, damage-initiating genes may need to be regulated fast, while late time points may rather reflect counter-regulations and secondary events (Balmer et al. 2014; Blaauboer et al. 2012). This is particularly true when tissues or complex 3D cell cultures (Alépée et al. 2014) are studied. In this case, it is not clear whether the transcriptome changes studied actually occur within a single cell or whether they are a composite reaction of the tissue (Gantner et al. 1996), consisting of different neuronal types plus microglia, activated astrocytes and possibly even invading blood cells. To play the devil’s advocate, SP-1 may not play a role in neurons, but rather in the activation—or the dampening—of the inflammatory response in glia, or it may be a late signal due to removal of tissue debris (Falsig et al. 2006; Hirt et al. 2000). Deconvolution of this complexity may require additional approaches and refinement, such as a multi-omics approach (Ramirez et al. 2013; Balmer and Leist 2014), or studies need to be limited to very early damage phases only. It is also still not clear, how this approach would best be applied in developmental toxicity, where the baseline of gene expression changes over time (Penschuk et al. 2006; Bal-Price et al. 2015; Smirnova et al. 2014). Finally, it needs to be considered, how far the changes observed in animals hold for gene regulatory networks in humans (Hartung and Leist 2008; Leist and Hartung 2013). A future example study in human cells would be an important complement to the present study.
